# Preclinical Evaluation of Novel Positron Emission Tomography (PET) Probes for Imaging Leucine-Rich Repeat Kinase 2 (LRRK2)

**DOI:** 10.1021/acs.jmedchem.3c01687

**Published:** 2024-02-02

**Authors:** Zhen Chen, Jiahui Chen, Wakana Mori, Yongjia Yi, Jian Rong, Yinlong Li, Erick R. Calderon Leon, Tuo Shao, Zhendong Song, Tomoteru Yamasaki, Hideki Ishii, Yiding Zhang, Tomomi Kokufuta, Kuan Hu, Lin Xie, Lee Josephson, Richard Van, Yihan Shao, Stewart Factor, Ming-Rong Zhang, Steven H. Liang

**Affiliations:** † Jiangsu Co-Innovation Center of Efficient Processing and Utilization of Forest Resources, Jiangsu Provincial Key Lab for the Chemistry and Utilization of Agro-Forest Biomass, Jiangsu Key Lab of Biomass-Based Green Fuels and Chemicals, International Innovation Center for Forest Chemicals and Materials, College of Chemical Engineering, Nanjing Forestry University, Nanjing, Jiangsu 210037, China; ‡ Department of Radiology and Imaging Sciences, Emory University, 1364 Clifton Rd, Atlanta, Georgia 30322, United States; § Division of Nuclear Medicine and Molecular Imaging, Massachusetts General Hospital & Department of Radiology, Harvard Medical School, Boston, Massachusetts 02114, United States; ∥ Department of Radiopharmaceuticals Development, National Institute of Radiological Sciences, National Institutes for Quantum and Radiological Science and Technology, Chiba 263-8555, Japan; ⊥ Department of Chemistry and Biochemistry, University of Oklahoma, Norman, Oklahoma 73019, United States; # Jean and Paul Amos Parkinson’s Disease and Movement Disorder Program, Department of Neurology, Emory University School of Medicine, Atlanta, Georgia 30322, United States

## Abstract

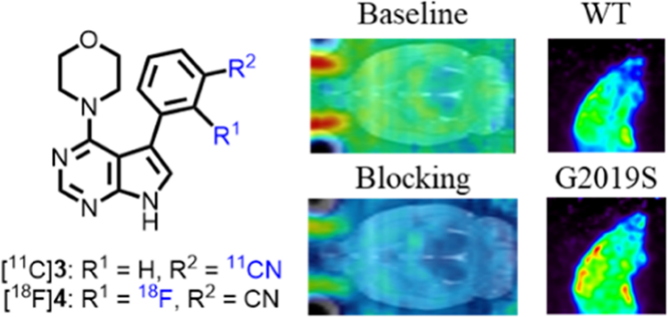

Parkinson’s disease (PD) is one of the most highly debilitating neurodegenerative disorders, which affects millions of people worldwide, and leucine-rich repeat kinase 2 (LRRK2) mutations have been involved in the pathogenesis of PD. Developing a potent LRRK2 positron emission tomography (PET) tracer would allow for in vivo visualization of LRRK2 distribution and expression in PD patients. In this work, we present the facile synthesis of two potent and selective LRRK2 radioligands [^11^C]**3** ([^11^C]PF-06447475) and [^18^F]**4** ([^18^F]PF-06455943). Both radioligands exhibited favorable brain uptake and specific bindings in rodent autoradiography and PET imaging studies. More importantly, [^18^F]**4** demonstrated significantly higher brain uptake in the transgenic LRRK2-G2019S mutant and lipopolysaccharide (LPS)-injected mouse models. This work may serve as a roadmap for the future design of potent LRRK2 PET tracers.

## 1 Introduction

Parkinson’s disease (PD) is a highly debilitating neurodegenerative disease that affects approximately 10% of people over 60 years old. Currently, there is no available disease-modifying therapy. Although remarkable investment has been devoted to many clinical trials to advance potential disease-modifying therapeutic intervention, the underlying disease mechanisms of PD still remain unclear, leading to significant clinical attrition.^1,2^ In past decades, several proteins have been identified for possible involvement in the pathogenesis of PD, and among them, leucine-rich repeat kinase 2 (LRRK2) represents a highly potential target.^3−5^ Indeed, LRRK2 mutations, which are autosomal dominant, represent the most common known cause of PD worldwide. Approximately 10% of inherited PD patients and 2% of sporadic cases carry LRRK2 mutations (e.g., G2019S, I2020T, R1441C), suggesting LRRK2 mutation likely as a common etiology underlying both inherited and sporadic PD.^6−9^ Notwithstanding growing evidence supporting the deep linkage of LRRK2 dysfunction with both inherited and non-inherited PD, it is still challenging to elucidate the physiological and/or pathological role of LRRK2 due to its complexity. Nevertheless, LRRK2 inhibitors are currently under investigation for the treatment of PD.^10^

**Figure 1:**
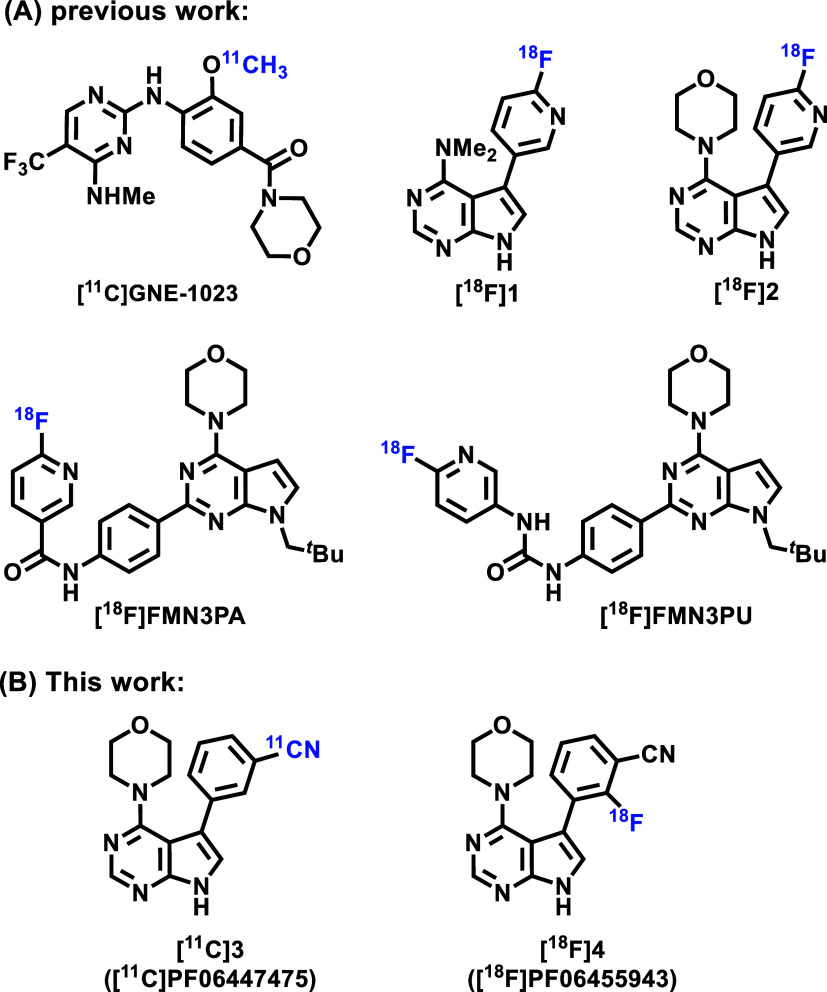
Representative LRRK2 PET tracers: (A) previous work; (B) this work.

Recently, noninvasive positron emission tomography (PET) has attained much attention in clinical trials, providing comprehensive visualization of in vivo biological processes and patient stratification for trial design and treatment efficacy evaluation.^11−13^ As part of our continuing efforts in LRRK2 inhibitor development, we are interested in identifying an appropriate LRRK2-selective PET agent.^14^ Indeed, an LRRK2-selective PET agent could enable the investigation of target engagement for a specific LRRK2 inhibitor, thus advancing its clinical characterization and translation. Moreover, in light of numerous pieces of evidence indicating increased LRRK2 enzyme activity in LRRK2 mutation-related PD brains, we are eager to investigate whether the LRRK2 distribution and expression in the brain of PD patients could be visualized by PET imaging, which would in turn give rise to a more in-depth understanding of PD pathogenesis. To date, several LRRK2 inhibitors have been labeled with carbon-11 or fluorine-18, which included GNE-1023^15^ and its analogues^16^ as well as HG-10-102-01,^17^ but limited biological data were disclosed (Figure 1A). As such, the development and validation of a blood–brain barrier (BBB)-penetrable LRRK2 PET tracer with excellent binding specificity are intensively motivated by the therapeutic potential of LRRK2 inhibitors. Toward this end, we were enlightened by PF-06447475 (**3**), an LRRK2 inhibitor lead initially disclosed by Pfizer.^14^ Through collaboration, we developed a promising fluorine-containing LRRK2 inhibitor lead PF-06455943 (**4**)^18^ by taking advantage of the central nervous system (CNS) PET radioligand design and multiparameter optimization (MPO) selection criteria.^19−22^ Both **3** and **4** have been comprehensively validated in pharmacology screening, revealing favorable pharmacological and pharmacokinetic characteristics such as excellent potency and target selectivity toward LRRK2, reasonable clearance rate, clean safety profile, high passive permeability, and low P-glycoprotein (P-gp) efflux (Table 1).^14,18^ Moreover, we successfully radiolabeled **4** with fluorine-18 and performed a preliminary PET imaging study of [^18^F]**4** ([^18^F]PF-06455943) in nonhuman primates (NHPs), which exhibited high brain uptake and binding specificity.^18^ During the course of preparing the current manuscript, based on the scaffold of **3**, Li et al.^23^ also developed two ^18^F-labeled LRRK2 PET ligands [^18^F]**1** and [^18^F]**2**, whereas Schaffer et al.^24^ disclosed [^18^F]FMN3PA and [^18^F]FMN3PU, despite lack of comprehensive pharmacological and pharmacokinetic information (Figure 1A). Therefore, we focus on developing an appropriate PET radioligand with comprehensively validated pharmacological and pharmacokinetic properties to enable preclinical and clinical characterization of LRRK2 inhibitors. In this work, we present herein a novel synthesis of radioligands [^11^C]**3** ([^11^C]PF-06447475) via a facile copper-mediated cyanation reaction and [^18^F]**4** via a nucleophilic S_N_Ar displacement reaction. Although the radiosynthesis of [^18^F]**4** and its preliminary evaluation in nonhuman primates (NHPs) have been described in our previous report, in this study, we aim to highlight the evaluation of [^11^C]**3** and [^18^F]**4** in rodent-based disease models. As a consequence, both radioligands demonstrated favorable brain uptake and specific bindings in rodents by autoradiography and PET imaging studies (Figure 1B). More importantly, in transgenic LRRK2-G2019S mutant and lipopolysaccharide (LPS)-injected mouse models, [^18^F]**4** exhibited significantly higher brain uptake compared to that of control mice. This work may serve as a roadmap for the future design of potent LRRK2 PET tracers.

**Table 1:** Comparison of the Pharmacological and Pharmacokinetic Properties of Representative LRRK2 Inhibitors^15,18,23,24^

compound	WT^a^ IC_50_ (nM)	G2019S^a^ IC_50_ (nM)	WCA^b^ IC_50_ (nM)	*K*_d_ (nM)	HLM CL^c^ (mL/min/kg)	THLE^d^ IC_50_ (μM)	MDR1^e^ BA/AB	RRCK^f^*P*_app_AB	*c *log *P*^g^	tPSA^g^
GNE-1023	2						1.2	18.2	2.13	87.55
**1**				6.7^h^					1.95	52.35
**2**				14.3^h^					1.24	61.58
FMN3PA				20^h^					4.51	81.89
FMN3PU				23.6^h^					5.15	93.92
**3**	3	14	53		36	>223	1.08	26.68	1.94	73.01
**4**	3.58	6.95	20		31.4	162	1.10	29.18	2.08	73.01

a Biochemical LRRK2 assays (*n* ≥ 2).

b Whole cellular LRRK2 assay (*n* ≥ 2); MDR1 efflux ratio.

c Human liver microsomal clearance.

d Transformed human liver epithelial (THLE) cell viability assay.

e MDR1 efflux ratio.

f Passive permeability as a rate in 1 × 10^–6^ cm/s.

g *c* log* P* and tPSA were calculated by ChemBioDraw Ultra 14.0 (CambridgeSoft Corporation, PerkinElmer).

h Saturation assays.

## 2 Results and Discussion

### 2.1 Molecular Docking Study

**Figure 2:**
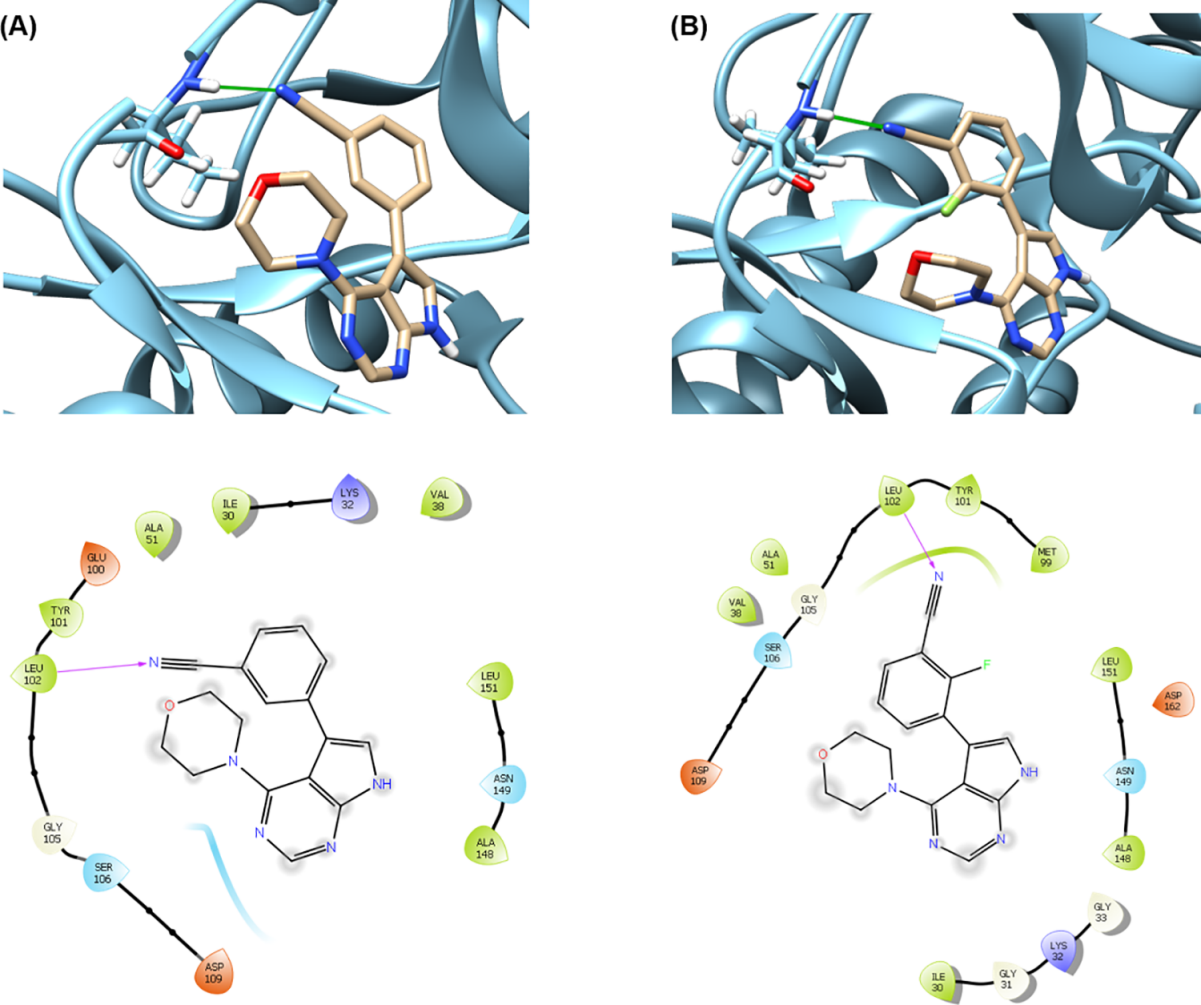
Molecular docking structures of compounds **3** (A) and **4** (B) onto MST3. The top insets at each panel exhibit the docking pose of each compound into the binding pocket. The bottom insets at each panel exhibit the significant hydrophobic and minor polar (light blue), glycine (white), and positively (dark blue) and negatively (red) charged interactions between each compound with the binding pocket. The PDB ID of the protein structure is4W8E.

To probe the possible molecular interactions of compounds **3** and **4** with LRRK2, a preliminary molecular docking study was carried out. Considering the similarity (73%) of the ATP-binding site residues between LRRK2 and mammalian STE20-like protein kinase 3 (MST3), an MST3-inhibitor complex was constructed as a surrogate for the LRRK2-inhibitor complex by Autodock Vina with PDB ID 4W8E as the building template. As shown in Figure 2, compounds **3** and **4** both fell into the binding pocket of MST3. Significant hydrophobic and minor polar, glycine, and charged interactions were observed between compounds **3** and **4** with the binding pocket. Notably, a hydrogen bond between the cyano N atom in compounds **3** and **4** with the Leu102 residue of the binding domain may exist, which highlighted the significance of the cyano group for the high binding potencies of these two compounds.

### 2.2 Radiochemistry

**Figure 3:**
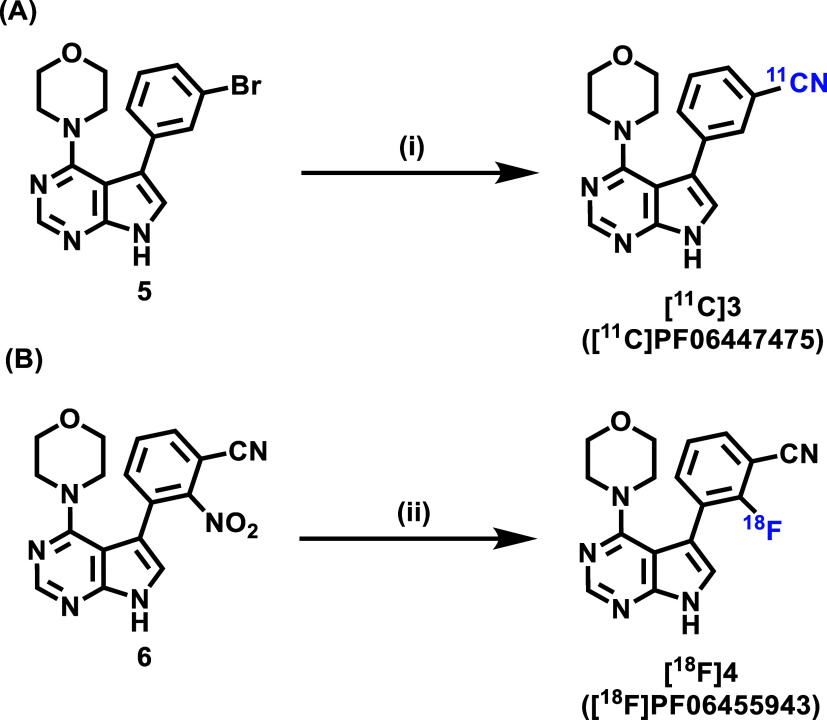
Preparation of radioligands [^11^C]**3** (A) and [^18^F]**4** (B). Conditions: (i) [^11^C]NH_4_CN, NH_4_HCO_3_, CuI, DMF, 180 °C, 5 min; 4.6% decay-corrected radiochemical yield; (ii) [^18^F]KF, K_2_CO_3_/K222, DMSO, 150 °C, 15 min; 18% nondecay-corrected radiochemical yield.

With promising pharmacological and pharmacokinetic results, we commenced the preparation of radioligands [^11^C]**3** and [^18^F]**4** for further investigation. As shown in Figure 3, the radioligand [^11^C]**3** ([^11^C]PF06447475) was synthesized via a copper-mediated cross-coupling reaction of the corresponding aryl bromide precursor **5** with [^11^C]cyanide, whereas the radioligand [^18^F]**4** was prepared via a S_N_Ar nucleophilic substitution reaction of the corresponding nitro precursor **6** with [^18^F]fluoride. Specifically, by heating the mixture of the bromide precursor **5**, [^11^C]cyanide, NH_4_HCO_3_, and CuI in *N*,*N*-dimethylformamide (DMF) at 180 °C for 5 min, the copper-mediated coupling reaction readily proceeded to produce [^11^C]**3** in 4.6% decay-corrected radiochemical yield (RCY). On the other hand, [^18^F]**4** was achieved in 18% RCY (nondecay-corrected) by reacting precursor **6** with [^18^F]fluoride and K_2_CO_3_/K222 in dimethyl sulfoxide (DMSO) at 150 °C for 15 min. Notably, for both radioligands [^11^C]**3** and [^18^F]**4**, excellent radiochemical purity and molar activity were obtained.

### 2.3 In Vitro Autoradiography

**Figure 4:**
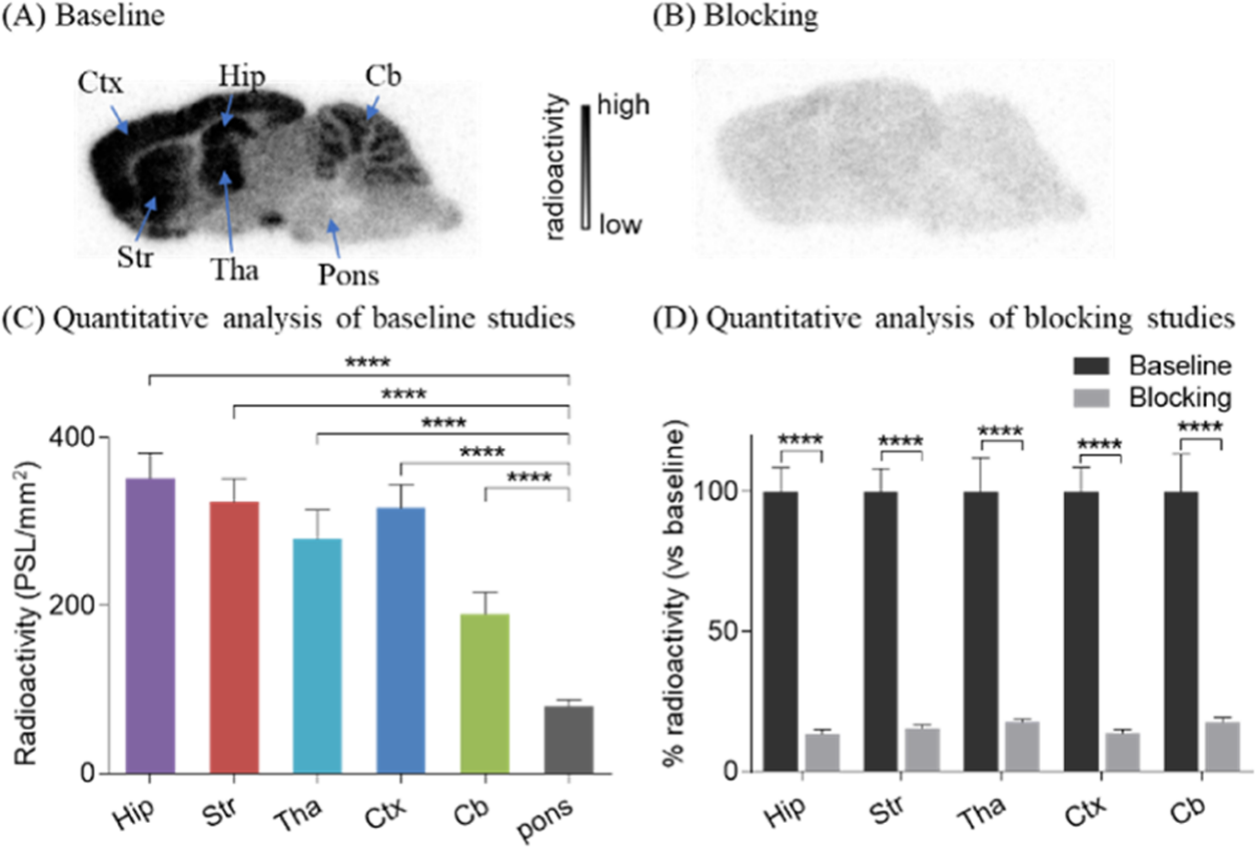
In vitro autoradiography of the radioligand [^11^C]**3** in rat brain sections. (A) Baseline; (B) blocking studies with **3** (10 μM); (C) quantification of radioactivity under baseline and blocking conditions. Hip = hippocampus; Tha = thalamus; Str = striatum; Ctx = cerebral cortex; and Cb = cerebellum. All data are mean ± SD, *n* = 3. Asterisks indicate the statistical significance. **p ≤ 0.01, ***p ≤ 0.001, and ****p ≤ 0.0001.

**Figure 5:**
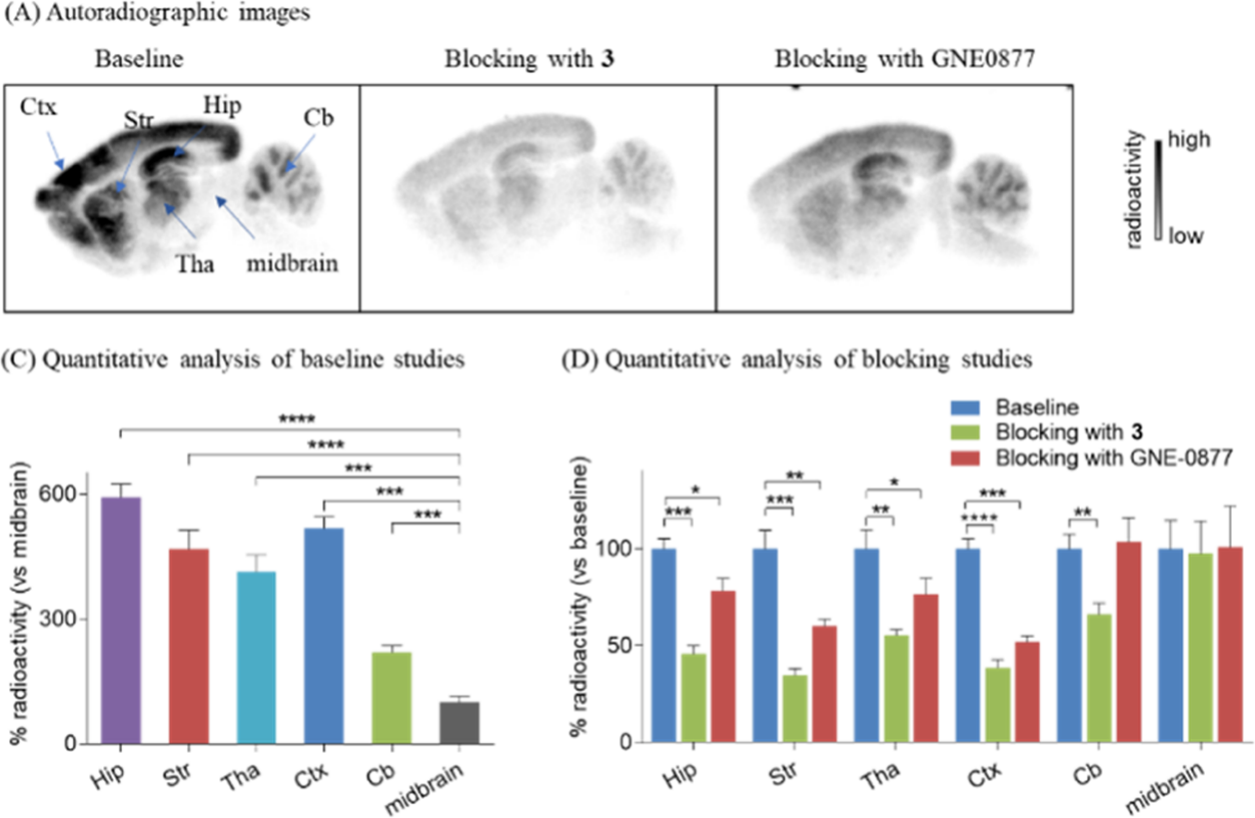
In vitro autoradiography of the radioligand [^18^F]**4** in rat brain sections. (A) Autoradiographic images under baseline, blocking with **3** (10 μM) and blocking with GNE-0877 conditions; (B) quantification of radioactivity under baseline conditions with the midbrain as the reference region; (C) quantification of radioactivity under blocking conditions. Hip = hippocampus; Tha = thalamus; Str = striatum; Ctx = cerebral cortex; Cb = cerebellum. All data are mean ± SD, *n* = 3. Asterisks indicate the statistical significance. *p < 0.05, **p ≤ 0.01, ***p ≤ 0.001, and ****p ≤ 0.0001.

To investigate the specific binding of [^11^C]**3** and [^18^F]**4** toward LRRK2, in vitro autoradiography was carried out in rat brain sections (Figures 4 and 5). As shown in Figure 4, in baseline studies, [^11^C]**3** revealed a heterogeneity of radioactivity levels in various rat brain regions. The highest uptake was seen in the hippocampus, followed by the striatum, cerebral cortex, thalamus, and cerebellum, and the lowest uptake was observed in the pons and midbrain. The radioactive distribution profile of [^11^C]**3** was in line with the LRRK2 expression pattern in rodents.^7,25,26^ Under self-blocking conditions (10 μM), radioactivity accumulations in all brain regions were remarkably reduced by 64–87%. Of particular note, brain regions featuring relatively high levels of LRRK2 exhibited much higher reduction of radioactive uptake, such as hippocampus (87%), striatum (85%), cerebral cortex (87%), thalamus (83%), and cerebellum (83%). By contrast, brain regions with low LRRK2 expression exhibited relatively lower reduced uptake of [^11^C]**3** (e.g., the pons, 64%). Similar to autoradiographic studies of [^11^C]**3**, [^18^F]**4** provided a heterogeneous distribution (hippocampus > cerebral cortex > striatum > thalamus > cerebellum > midbrain) under baseline conditions as well as remarkably decreased uptake (34–65%) when compound **3** was used in the pretreatment studies (Figure 5), which was in line with the results from NHP autoradiographic studies.^18^ To further demonstrate the binding specificity of [^18^F]**4**, a structurally diverse blocking reagent GNE-0877 was used in autoradiographic studies. As shown in Figure 5, the radioactivity levels in LRRK2-rich brain regions (hippocampus, cerebral cortex, striatum, and thalamus) were significantly reduced, whereas no obvious blocking results were observed in LRRK2-deficient brain regions (cerebellum and midbrain). These results indicated excellent in vitro specific binding of both [^11^C]**3** and [^18^F]**4** toward LRRK2.

### 2.4 Preliminary Rat PET Imaging

**Figure 6:**
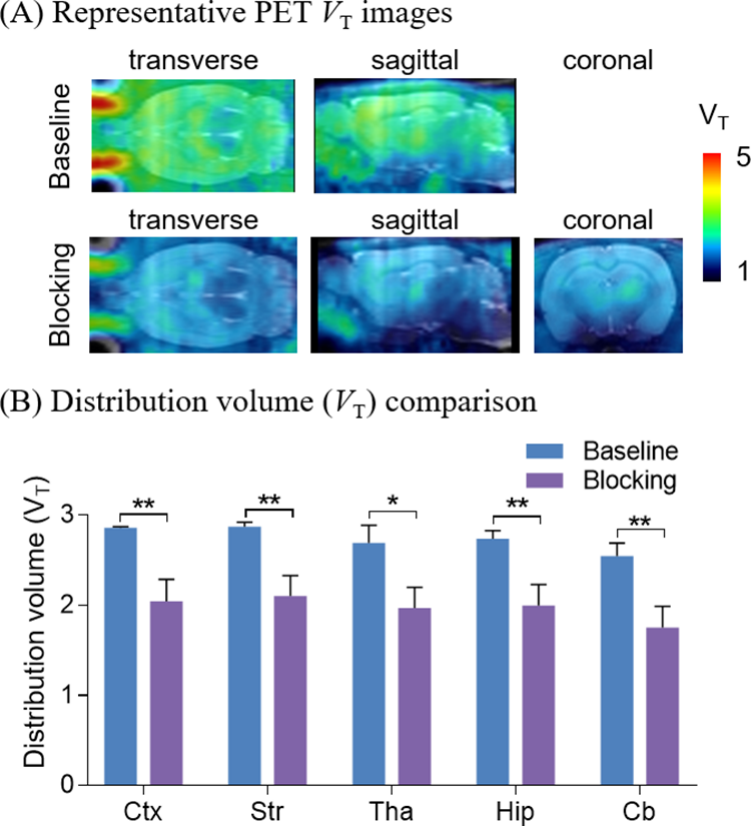
PET images of [^18^F]**4** in rats. (A) Representative PET images under baseline and blocking conditions; (B) distribution volume (*V*_T_) comparison between baseline and blocking conditions. Hip = hippocampus; Tha = thalamus; Str = striatum; Ctx = cerebral cortex; Cb = cerebellum. Asterisks indicate the statistical significance. *p < 0.05, and **p ≤ 0.01.

The promising pharmacological, pharmacokinetic, and in vitro autoradiographic data encouraged us to carry out preliminary dynamic PET imaging studies of [^11^C]**3** and [^18^F]**4**. As shown in Figure S1, both ligands rapidly penetrated the BBB, and the heterogeneous distribution pattern of radioactive signals in rat brains paralleled well with results from in vitro autoradiography and LRRK2 mRNA expression in rodents as previously reported.^26−28^ Given the superiority of fluorine-18 such as clean positron emission, low positron range, and relatively long half-life compared with carbon-11, [^18^F]**4** was subjected to further investigation (Figure 6). A blocking scan was carried out with intravenous administration of compound **3** (1 mg/kg) prior to tracer injection. For both baseline and blocking studies, blood samples were extracted from the artery. Both whole-blood and plasma radioactivity concentrations were evaluated, and radiometabolites were measured in plasma samples by radio high-performance liquid chromatography (HPLC) to generate metabolite-corrected input function (Figure S2). Compartmental analyses with one- and two-tissue compartment models (1TCM and 2TCM) were carried out on regional time–activity curves. A 2TCM with reversible binding exhibited better fits to all brain regions with a stable volume of distribution (*V*_T_). In the baseline study, *V*_T_ of various brain regions ranged from 2.2 to 2.9 mL/cm^3^, confirming the high binding of the radiotracer. Pretreatment of compound **3** resulted in a significant decrease of uptake in various brain regions, which suggested encouraging binding specificity of [^18^F]**4**. Furthermore, reasonable in vivo metabolic stability of [^18^F]**4** was also demonstrated with 38% and 23% parent fractions in the plasma of rats at 30 and 60 min post tracer administration, respectively (Figure S2).

### 2.5 Ex Vivo Biodistribution

**Figure 7:**
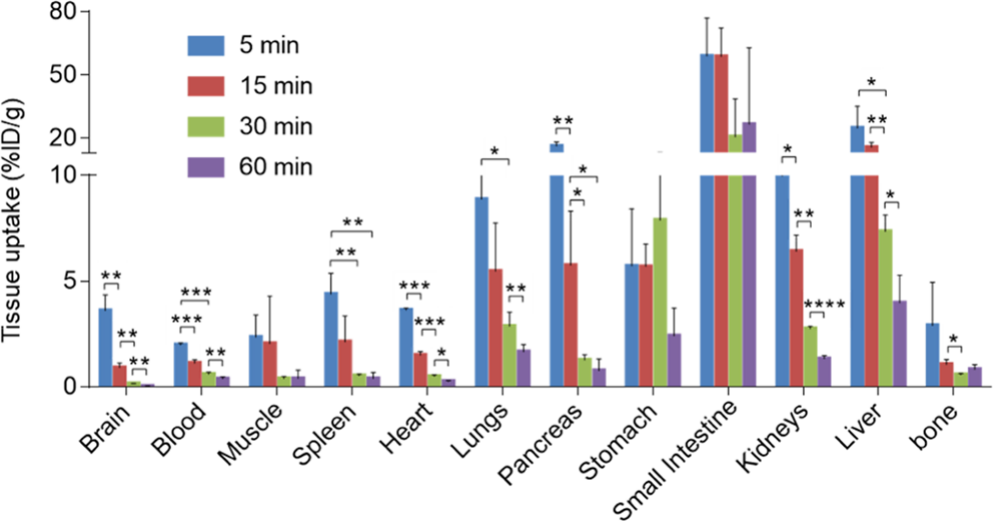
Whole-body ex vivo biodistribution studies. Asterisks indicate the statistical significance. *p < 0.05, **p ≤ 0.01, ***p ≤ 0.001, and ****p ≤ 0.0001. %ID/g = injected dose per gram of tissue.

In the present study, [^18^F]**4** was used for further evaluation due to its superior RCY and high molar activity, longer half-life, and improved brain distribution profile compared with that of [^11^C]**3**. With the established good BBB penetration ability of [^18^F]**4**, we then performed ex vivo biodistribution studies to obtain more in-depth information on the whole-body distribution and clearance of [^18^F]**4**. Mice were sorted into four groups based on their survival intervals (5, 15, 30, and 60 min) after intravenous administration of [^18^F]**4**. As shown in Figure 7 and Table S2, initial high radioactive signals were seen in several peripheral organs such as the spleen, heart, lungs, pancreas, stomach, small intestine, kidneys, and liver (>4%ID/g, injected dose per gram of tissue), followed by rapid clearance in almost all of these organs except the stomach and small intestine. The slow radioactivity clearance in the small intestine, together with high radioactive signals in the small intestine and liver at 60 min post tracer injection, suggested the hepatobiliary and urinary elimination pathway of [^18^F]**4**. Additionally, no remarkable de-radiofluorination was seen during the current study. To investigate the in vivo stability of [^18^F]**4** in mouse brains, we conducted a radiometabolic analysis in mouse brain homogenate at 30 min post tracer administration. The metabolism in the mouse brain was found to be reasonable with the parent fraction of 69% (Figure S3).

### 2.6 PET Imaging of [^18^F]**4** in Disease Models

**Figure 8:**
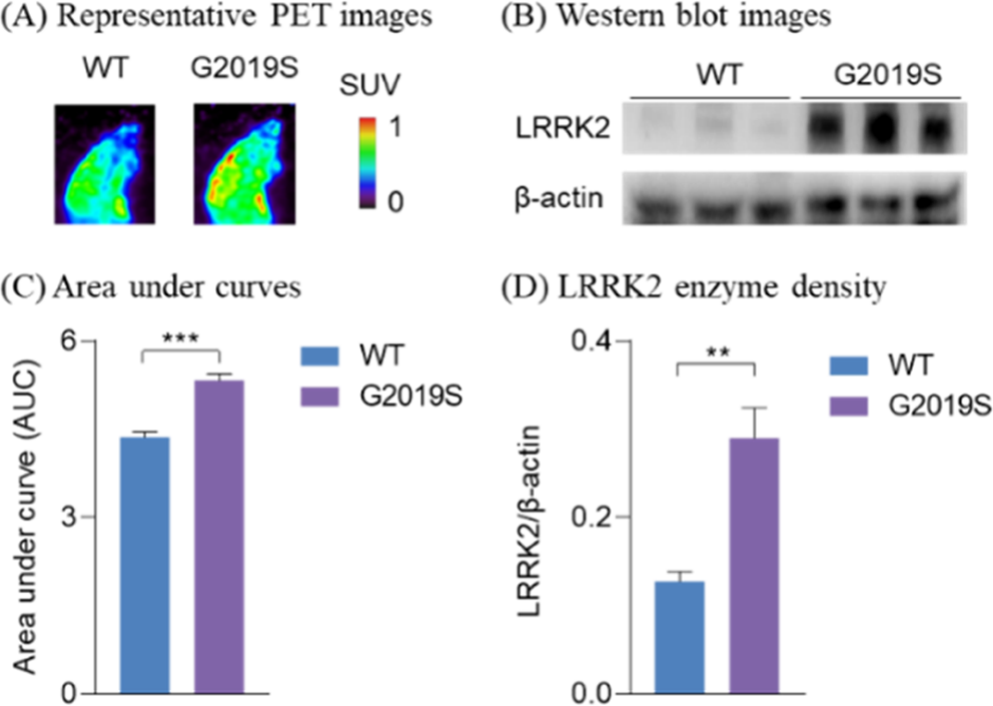
Validation of [^18^F]**4** in LRRK2-G2019S mutant and wild-type (WT) mice. (A) Representative PET images (0–10 min summed); (B) representative Western blot images; (C) quantitative analysis of area under curves for PET imaging studies; and (D) quantitative analysis of LRRK2 enzyme density. Asterisks indicate the statistical significance. **p ≤ 0.01 and ***p ≤ 0.001.

**Figure 9:**
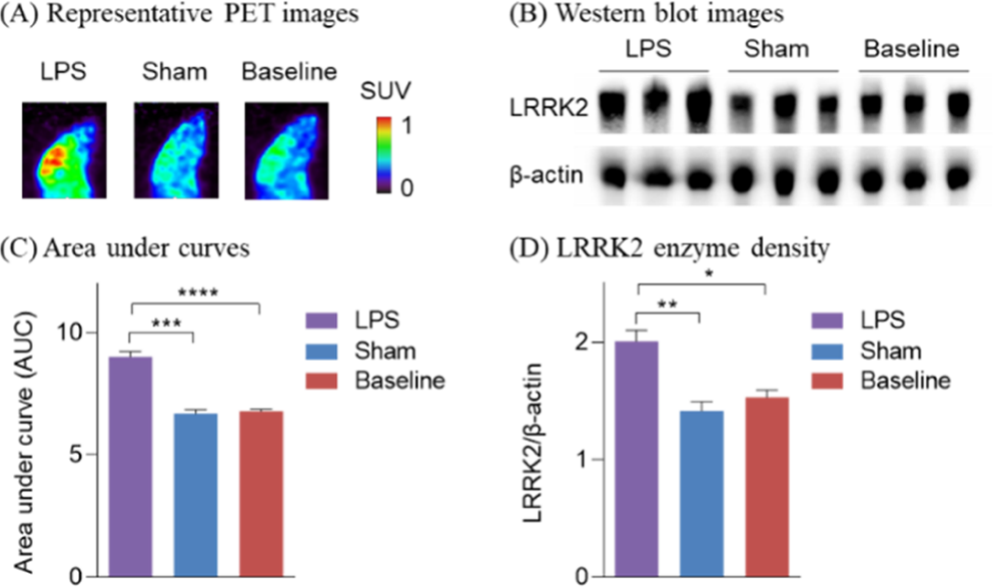
Validation of [^18^F]**4** in LPS mice. (A) Representative PET images in LPS, sham, and baseline mice (0–15 min summed); (B) representative Western blot images. Quantitative analysis of (C) area under curves and (D) LRRK2 enzyme density. Asterisks indicate the statistical significance. *p ≤ 0.05, **p ≤ 0.01, ***p ≤ 0.001, and ****p ≤ 0.0001.

To further showcase the translational feasibility of [^18^F]**4**, we carried out dynamic PET scans in model mice with the transgenic LRRK2-G2019S mutant and the corresponding wild-type (WT) mice. It is worth mentioning that G2019S is a dominant LRRK2 mutation, which replaces the glycine at amino acid 2019 with serine. Studies have demonstrated that G2019S leads to an increased LRRK2 kinase activity.^29^ Furthermore, G2019S has also proved as related to not only familial and sporadic PD but also impairment of adult neurogenesis in mice.^30,31^ As shown in Figure 8, [^18^F]**4** rapidly accumulated in both LRRK2-G2019S and WT mouse brains within 3 min, followed by rapid elimination. [^18^F]**4** revealed statistically significant higher brain uptake in G2019S mice compared with that of WT mice. Quantitative analysis indicated that there was around 22% increase (*p* ≤ 0.001) of radioactivity accumulation based on the area under curve (AUC) in G2019S mice. Moreover, we measured ex vivo LRRK2 expression levels in both G2019S and WT mouse brains by Western blot, which demonstrated a 2.26-fold increase of LRRK2 enzymes in G2019S mouse brains. These results suggested that the increased uptake of [^18^F]**4** was consistent with the increased LRRK2 enzyme expression in LRRK2-G2019S mouse models, although in vivo PET results are not as profound as those obtained from in vitro Western blot analysis. Considering the significant involvement of LRRK2-G2019S mutation in PD, [^18^F]**4** may represent a promising PET ligand for studying LRRK2 changes in PD. Additionally, emerging evidence has supported that neuroinflammation is often associated with PD pathogenesis and could be attenuated by LRRK2 blockade.^32−34^ As a proof of concept, we utilized a neuroinflammatory mouse model by intracranial injection of LPS and conducted preliminary PET imaging studies with [^18^F]**4**. As shown in Figure 9, the LPS-injected mice revealed much higher brain uptake of [^18^F]**4** compared to the sham group injected with phosphate-buffered saline (PBS), with 28% increase of radioactivity based on the AUCs, which is consistent with the Western blot results. This preliminary result built a foundation for the feasibility of probing LRRK2 changes in neuroinflammation rodent models.

## 3 Conclusions

Previously, PF-06447475 (**3**) and PF-06455943 (**4**) were identified as two highly potent LRRK2 inhibitors. Both compounds feature favorable pharmacological and pharmacokinetic characteristics, such as excellent binding affinity and target selectivity toward LRRK2, reasonable clearance rate, clean safety profile, high passive permeability, low P-gp efflux, and favorable unbound free fraction in the brain and plasma. As a step forward for our continuing interest in LRRK2 PET ligand development, we present herein our successful synthesis of two LRRK2 PET ligands via a copper-mediated cyanation reaction for [^11^C]**3** ([^11^C]PF-06447475) and base-promoted nucleophilic S_N_Ar displacement reaction for [^18^F]**4** ([^18^F]PF-06455943). Both radioligands exhibited favorable radiochemical yields, excellent radiochemical purities, and good molar activities. Further evaluation of [^11^C]**3** and [^18^F]**4** by autoradiography and PET imaging studies in rodents demonstrated good brain uptake and favorable specific bindings. It is noteworthy that [^18^F]**4** exhibited higher brain uptake in the G2019S mutant and LPS-injected mice compared with that of control mice. Taken together, [^18^F]**4** may represent a novel promising PET tracer for studying LRRK2 changes during PD progression, which thereafter warrants more comprehensive preclinical and clinical validations.

## 4 Experimental Section

The experimental procedures used in this work were slightly modified from the literature.^35−38^ All of the chemicals used in the synthesis of LRRK2 inhibitors and the corresponding precursor were directly acquired from commercial vendors without any purification. Silica gel was used for the purification of synthetic compounds by column chromatography, and 0.25 mm silica gel plates were used as indicators for TLC. All heating reactions were heated by a metal sand bath (WATTCAS, LAB-500). To obtain the NMR spectra of synthetic compounds, a 300 MHz Bruker spectrometer was used. “ppm” was used to indicate the chemical shifts (δ), and “hertz” was the unit of coupling constants. The abbreviations of multiplicities for peaks in the HNMR and FNMR spectra were described as follows: s (singlet), d (doublet), dd (doublet of doublets), t (triplet), q (quartet), m (multiple), and br (broad signal). For the measurement of mass spectrometry, an Agilent 6430 Triple Quad LC/MS was adopted with ESI as the ionization approach. No promiscuity was observed in the assay of PAINS (Pan Assay Interference Compounds) for compounds **3** and **4** with two in silico filters (http://zinc15.docking.org/patterns/homeandhttp://www.swissadme.ch/index.php).^39^ High purity (≥95%) was also determined for compounds **3** and **4** by reverse-phase HPLC (Agilent 5 μm, Eclipse plus C18 column (4.6 mm ID × 100 mm)). Unless otherwise stated, molar activity was determined at the end of the synthesis. All animal studies were carried out following the ethical rules of our institutional policy. CD-1 mice (female, 22**–**24 g, 7 weeks), Sprague–Dawley (SD) rats (male, 210**–**230 g, 7–9 weeks), LRRK2-G2019S mutation knock-in mice, and wild-type mice (female, 25–28 g, 6–7 months) were fed ad libitum with food and water under a 12 h light/12 h dark cycle condition.

### 4.1 Radiosynthesis of [^11^C]**3**

[^11^C]HCN was yielded from cyclotron-produced [^11^C]CO_2_ by the ^14^N(p, α)^11^C nuclear reaction. In brief, [^11^C]CO_2_ was first converted to [^11^C]CH_4_ with H_2_ on Ni at 400 °C and then to [^11^C]HCN with NH_3_ on Pt at 900 °C. He was used as a carrier gas. The [^11^C]HCN was trapped in a solution of NH_4_HCO_3_ in 1.7 mL of water (0.32 g/mL). We measured the amount of [^11^C]CN that is captured by attaching the vent line of the reaction vial to a charcoal trap. Passing the [^11^C]HCN gas mixture through NH_4_HCO_3_ aqueous solution leads to an approximately 150 mCi of [^11^C]HCN captured in solution at 10 min post bombardment. The ammonium [^11^C]cyanide solution obtained was transferred to a 1.5 mL reaction vial containing the precursor **5** (2.0 mg), CuI (1.2 mg), and anhydrous DMF (300 μL), and the reaction mixture was agitated at 180 °C for 5 min.

After cooling to room temperature, the reaction mixture was then diluted with the HPLC mobile phase (3.5 mL), followed by the injection into an HPLC column. HPLC purification was performed on a COSMOSIL Cholester column (10 mm × 250 mm, 5 μm) using a mobile phase of CH_3_CN/0.1 M NH_4_OAc (60/40) at a flow rate of 4.5 mL/min. The reaction time of [^11^C]**3** was 5.1 min. The radioactive fraction corresponding to the desired product was collected in a sterile flask, evaporated to dryness in vacuo, and reformulated in a saline solution (3 mL) containing 100 μL of 25% ascorbic acid in sterile water and 100 μL of 20% Tween 80 in ethanol. The synthesis time was 70 min from the end of bombardment. Radiochemical and chemical purity were measured by analytical HPLC COSMOSIL Cholester column (4.6 mm × 250 mm, 5 μm) using a mobile phase of CH_3_CN/0.1 M NH_4_OAc (60/40) at a flow rate of 1.0 mL/min. The identity of [^11^C]**3** was confirmed by the coinjection with unlabeled **3**. The radiochemical yield was 4.6% nondecay-corrected based on [^11^C]CO_2_ with >99% radiochemical purity, and the molar activity was 2.5 Ci/μmol.

### 4.2 Radiosynthesis of [^18^F]**4**

The general procedure was described previously.^18^ The cyclotron-produced [^18^F]HF (approximately 500 mCi) was separated from H_2_^18^O using a Sep-Pak Accell Plus QMA Plus Light cartridge (Waters; Milford, Ma). The produced [^18^F]HF was eluted from the cartridge with a solution of K_2_CO_3_ (3 mg) and K222 (15 mg) in water (300 μL) and CH_3_CN (700 μL), and transferred to a reaction vessel in the hot cell as [^18^F]KF. After drying the [^18^F]KF solution at 150 °C for 30 min to remove water and CH_3_CN, a solution of nitro precursor **6** (1.5 mg) in anhydrous DMSO (700 μL) was then added. The vessel was heated at 150 °C for 15 min and then diluted with an HPLC mobile phase (3.5 mL), followed by injection into an HPLC column. HPLC purification was performed on an X-Select Prep C18 column (10 mm × 250 mm, 5 μm) using a mobile phase of CH_3_CN/0.1 M ammonium formate (AMF) (30/70) at a flow rate of 5.0 mL/min. The retention time of [^18^F]**4** was 16.9 min. The radioactive fraction corresponding to the desired product was collected in a sterile flask, diluted with 30 mL of water, and trapped on a Sep-Pak light HLB cartridge. After washing with 10 mL of water to remove the CH_3_CN residue, the product was washed out from the cartridge with 1 mL of ethanol and formulated with 10 mL of phosphate-buffered saline (PBS). The synthesis time was 70 min from the end of bombardment. Radiochemical and chemical purity were measured by analytical HPLC Gemini NX-C18 column (3 mm × 150 mm, 5 μm) using a mobile phase of CH_3_CN/0.1 M AMF (30/70) at a flow rate of 0.8 mL/min. The identity of [^18^F]**4** was confirmed by the coinjection with unlabeled **4**. The radiochemical yield was 18% nondecay-corrected based on [^18^F]F^–^ with >99% radiochemical purity, and the molar activity was greater than 1.0 Ci/μmol.

### 4.3 In Vitro Autoradiography

The general procedure for autoradiography studies was described previously with minor revision in this work.^36,40^ Brain sections from rats were preincubated with Tris-HCl buffer (50 mM), MgCl_2_ (1.2 mM) and CaCl_2_ (2 mM) solution for 20 min at ambient temperature, followed by incubation with [^11^C]**3** and [^18^F]**4** (0.48 nM). For blocking studies, PF-06447475 (10 μM), a known LRRK2 inhibitor, was added to the incubation solution in advance to determine the specificity of radioligand binding. After incubation, brain sections were rinsed with ice-cold buffer 3 times for 2 min and dipped in cold distilled water for 10 s. The brain sections were dried with cold air and then placed on imaging plates (BAS-MS2025, GE Healthcare, NJ) for optimized contact periods. Autoradiograms were obtained and regions of interest (ROIs) were carefully drawn with the reference of naked-eye observation. Radioactivity was measured using an Amersham Typhoon 5 analyzer system and expressed as photostimulated luminescence values per unit area (PSL/mm^2^) or normalized to % of radioactivity vs control.

### 4.4 PET Imaging in Rats

The general procedure for PET studies was described previously^35,41^ with minor modification in this work. Briefly, PET scans were carried out with an Inveon PET scanner (Siemens Medical Solutions, Knoxville, TN). Sprague–Dawley rats were kept under anesthesia using 1–2% (v/v) isoflurane during the scan. The radiotracer (ca. 0.5 mCi/150 μL) was injected into the tail vein via a preinstalled catheter. A dynamic scan in the three-dimensional (3D) list mode was acquired for 60 min. For pretreatment studies, a solution of PF06447475 (3 mg/kg) in 300 μL saline containing 10% ethanol and 5% Tween 80 was injected via the pre-embedded tail vein catheter at 30 min prior to tracer injection. As we previously reported,^40−42^ the PET dynamic images were reconstructed using ASIPro VW software (Analysis Tools and System Setup/Diagnostics Tool, Siemens Medical Solutions). Volumes of interest, including the hippocampus, cortex, cerebellum, striatum, and thalamus, were placed using ASIPro software. The radioactivity was decay-corrected and expressed as the standard uptake value: SUV = (radioactivity per mL tissue/injected radioactivity) × body weight.

### 4.5 Ex Vivo Whole-Body Biodistribution of [^18^F]**4** in Mice

The general procedure for ex vivo biodistribution studies was described previously^35,41^ with minor modification in this work. Briefly, a solution of [^18^F]**4** (50 μCi/100 μL) was injected into CD-1 mice via tail vein. These mice (each time point *n* = 4) were sacrificed at 5, 15, 30, and 60 min post tracer injection. Major organs, including whole brain, heart, liver, lung, spleen, kidneys, small intestine (including contents), muscle, and blood samples, were quickly harvested and weighted. The radioactivity present in these tissues was measured using a Cobra Model 5002/5003 γ counter, and all radioactivity measurements were automatically decay-corrected based on the half-life of fluorine-18. The results are expressed as the percentage of injected dose per gram of wet tissue (%ID/g).

### 4.6 Animals and Treatments

The CD-1 mice were anesthetized and secured in a stereotaxic frame. The skull was exposed and stereotaxic coordinates (−2.6 mm dorsal/ventral, −1.5 mm lateral, and −0.2 mm anterior/posterior from bregma) according to the procedure by Haley and McCormick.^43^ The i.c.v. injections of 10 μg (in 2 μL of saline) of LPS and saline (control group) injections were administered using a microsyringe. The behavior of each mouse was characterized and recorded in the form of scores, including appearance, activity, level of consciousness, eyes, respiration rate, and respiration quality. Scores were determined by summing up the individual scores (Figure S10).

### 4.7 Western Blot Analysis

Mouse brain tissues were homogenized using RIPA lysis buffer (Thermo Scientific, GA) supplemented with protease inhibitor (Thermo Scientific, GA). The homogenate was then centrifuged at 12,000*g* for 20 min at 4 °C. Equal amounts of protein from different experimental groups were subsequently separated via sodium dodecyl sulfate–polyacrylamide gel electrophoresis (SDS–PAGE) and transferred onto a nitrocellulose membrane. Afterward, the membranes were blocked for 1 h using a 5% skim milk solution and incubated with LRRK2 primary antibody (ab133474, 1:1000 dilution, Abcam, MA) or β actin antibody (ab115777, 1:1000 dilution, Abcam, MA) overnight at 4 °C. Then, the membranes were incubated in a horseradish peroxidase-conjugated secondary antibody (A16096, 1:2000 dilution Thermo Scientific, GA) for 1 h at room temperature. The signals were detected using an enhanced chemiluminescence kit (1705061, Bio-Rad) with a ChemiDoc imaging system (Bio-Rad, MA), and the results were analyzed using image lab software.

### 4.8 PET Imaging in Mouse Models

The general procedure for PET studies was described previously^35,41^ with minor modification in this work. Briefly, PET scans were carried out by a Genisys 4 PET scanner (Sofie Biosciences, Culver, CA). Mice were kept under anesthesia using 1–2% (v/v) isoflurane during the scan. The radiotracer (ca. 45 μCi/100 μL) was injected into the tail vein via a preinstalled catheter. A dynamic scan in 3D list mode was acquired for 60 min. The PET dynamic images were reconstructed using G4 software (Analysis Tools and System Setup/Diagnostics Tool, Sofie Biosciences). The radioactivity was decay-corrected and expressed as the standardized uptake value: SUV = (radioactivity per mL tissue/injected radioactivity) × body weight.

## Data Availability

The article contains the complete data used to support the findings of this study.

## Supporting Information Available

Experimental procedures; compound characterization data; computation details, and ^1^H, ^19^F, and ^13^C NMR spectra for new compounds (PDF)
Molecular formula strings (CSV)
Prot2_c4_complex (PDB)
Prot2_c3_complex (PDB)

## Supplementary Material

jm3c01687_si_002.csv

jm3c01687_si_003.pdb

jm3c01687_si_004.pdb

## Abbreviations

AUC: area under curve
BBB: blood–brain barrier
CNS: central nervous system
DMF: *N*,*N-*dimethylformamide
DMSO: dimethyl sulfoxide
%ID/g: injected dose per gram of tissue
LRRK2: leucine-rich repeat kinase 2
LPS: lipopolysaccharide
MPO: multiparameter optimization
MST3: mammalian STE20-like protein kinase 3
NHP: nonhuman primate
PD: Parkinson’s disease
PET: positron emission tomography
P-gp: P-glycoprotein
RCY: radiochemical yield
TCM: tissue compartment model
*V*_T_: volume of distribution
WT: wild type
