# Supplementary material for: Preclinical Evaluation of Novel Positron Emission Tomography (PET) Probes for Imaging Leucine-Rich Repeat Kinase 2 (LRRK2)
Source: J Med Chem. 2024 Feb 2;67(4):2559–69. doi: 10.1021/acs.jmedchem.3c01687 (PMC10895652; doi:10.1021/acs.jmedchem.3c01687)
Supplement: Supplementary file 1 — jm3c01687_si_001.pdf [file jm3c01687_si_001.pdf]

Supporting Information

Preclinical evaluation of novel positron emission tomography (PET) probes for imaging leucine-rich repeat kinase 2 (LRRK2)

Zhen Chen,[1,3,#] Jiahui Chen,[2,3#] Mori Wakana,[4,#] Yongjia Yi,[1] Jian Rong, [2,3] Yinlong Li,[2,3] Erick R. Calderon Leon,[5] Tuo Shao,[3] Zhendong Song,[2] Tomoteru Yamasaki,[4] Hideki Ishii[4], Yiding Zhang,[4] Tomomi Kokufuta[4], Kuan Hu[4], Lin Xie[4], Lee Josephson,[3] Richard S. Van,[5] Yihan Shao,[5] Stewart Factor,[6] Ming-Rong Zhang,[4,*] and Steven H. Liang[2,3]*

[1]Jiangsu Co-Innovation Center of Efficient Processing and Utilization of Forest Resources, Jiangsu Provincial Key Lab for the Chemistry and Utilization of Agro-Forest Biomass, Jiangsu Key Lab of Biomass-Based Green Fuels and Chemicals, International Innovation Center for Forest Chemicals and Materials, College of Chemical Engineering, Nanjing Forestry University, Nanjing, Jiangsu, 210037, China.

[2] Department of Radiology and Imaging Sciences, Emory University, 1364 Clifton Rd, Atlanta, Georgia 30322, United States.

[3]Division of Nuclear Medicine and Molecular Imaging, Massachusetts General Hospital & Department of Radiology, Harvard Medical School, Boston, MA, 02114, United States.

[4]Department of Radiopharmaceuticals Development, National Institute of Radiological Sciences, National Institutes for Quantum and Radiological Science and Technology, Chiba 263-8555, Japan

[5]Department of Chemistry and Biochemistry, University of Oklahoma, Norman, Oklahoma 73019, United States

Corresponding Author

* For M.R.Z.: Tel: +81 433 823 709. Fax: +81-43-206-3261. E-mail: zhang.ming-rong@qst.go.jp.

* For S.H.L.: Tel: +1 617 726 6107. E-mail: steven.liang@emory.edu.

Table of Contents

1. Supporting Scheme, table and figures S3

Table S1. Pharmacological and pharmacokinetic properties of PF-06447475 and PF-06455943[1] S3

Table S2. Ex vivo biodistribution of [^{18}F]**4** in mice. Data are expressed as %ID/g (mean ± SD, n = 4). S3

Figure S1. Baseline PET imaging studies of [^{11}C]**3** (A) and [^{18}F]**4** (B). S3

Figure S2. PET study of [^{18}F]**4** with blood sampling. (A) Representative PET images; (B) Plasma input curve and time-activity curves of [^{18}F]**4** in brain regions; (C) Distribution volume (V_T) comparision. S4

Figure S3. Metabolic analysis of [^{18}F]**4** in mouse brain at 30 min post tracer injection. S4

Figure S4. HPLC traces for [^{11}C]**3**. S5

Figure S5. HPLC trace for **3**. S5

Figure S6. HPLC traces for [^{11}C]**3** co-injected with **3**. S5

Figure S7. HPLC traces for [^{18}F]**4**. S5

Figure S8. HPLC trace for **4**. S6

Figure S9. HPLC traces for [^{18}F]**4** co-injected with **4**. S6

Figure S10. Disease scores of the LPS-induced mouse model every 72 hours (n = 4). S6

Figure S11. All heating reactions were heated by a metal sand bath (WATTCAS, LAB-500). S6

Scheme S1. Synthesis of standard LRRK2 inhibitors **3**&**4** and their radiolabeling precursors **10**&**11**.[1, 2] S7

2. NMR Spectra S10

3. References S11

1. Supporting Scheme, table and figures

Table S1. Pharmacological and pharmacokinetic properties of PF-06447475 and PF-06455943[1]

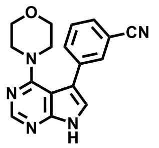

3 (PF-06447475)

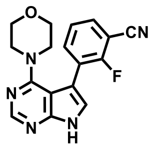

4 (PF-06455943)

Cpd	WT IC_{50} (nM)	G2019S IC_{50} (nM)	WCA IC_{50} (nM)	HLM CL (mL/min/kg)	THLE IC_{50} (μM)	MDR1 BA/AB	RRCK P_{app}AB
**3**	3	14	25	36	>223	1.0	27
**4**	3	9	20	31.4	162	1.1	29.2

Table S2. Ex vivo biodistribution of [^{18}F]**4** in mice. Data are expressed as %ID/g (mean ± SD, n = 4).

tissue	5 min	15 min	30 min	60 min
**brain**	3.61 ± 0.74	0.91 ± 0.21	0.14 ± 0.01	0.04 ± 0.01
**blood**	1.98 ± 0.08	1.12 ± 0.16	0.60 ± 0.08	0.37 ± 0.09
**muscle**	2.34 ± 1.06	2.04 ± 2.24	0.38 ± 0.11	0.39 ± 0.40
**spleen**	4.38 ± 0.99	2.12 ± 1.24	0.52 ± 0.07	0.39 ± 0.29
**heart**	3.63 ± 0.10	1.49 ± 0.17	0.49 ± 0.07	0.25 ± 0.03
**lungs**	8.88 ± 2.99	5.46 ± 2.28	2.86 ± 0.67	1.65 ± 0.35
**pancreas**	16.19 ± 1.99	5.75 ± 2.56	1.26 ± 0.25	0.76 ± 0.55
**stomach**	5.72 ± 2.69	5.69 ± 1.06	7.88 ± 5.2	2.41 ± 1.31
**small intestine**	58.87 ± 18.10	58.62 ± 13.63	20.55 ± 17.97	26.44 ± 36.34
**kidneys**	9.96 ± 1.01	6.41 ± 0.76	2.74 ± 0.12	1.32 ± 0.15
**liver**	24.68 ± 10.36	15.53 ± 2.40	7.34 ± 0.78	3.97 ± 1.30
**bone**	2.89 ± 2.05	1.06 ± 0.23	0.57 ± 0.05	0.82 ± 0.22

(A) Representative PET images and time–activity curves of [^{11}C]**3** in rat brain

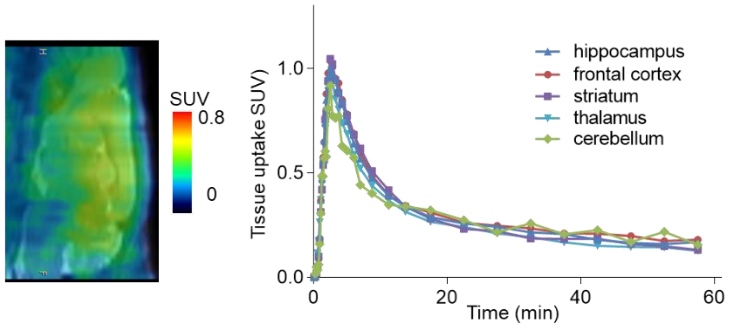

(B) Representative PET images and time–activity curves of [^{18}F]**4** in rat brain

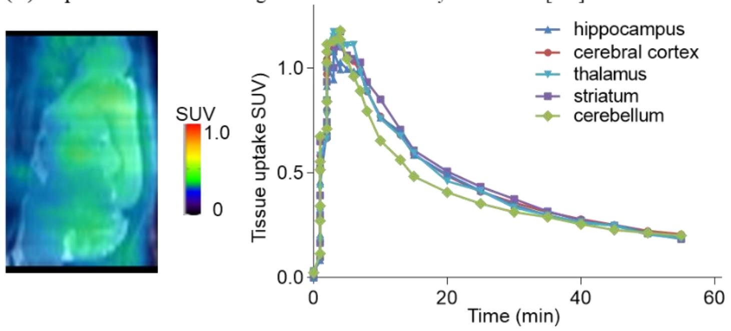

Figure S1. Baseline PET imaging studies of [^{11}C]**3** (A) and [^{18}F]**4** (B).

(A) Representative PET images

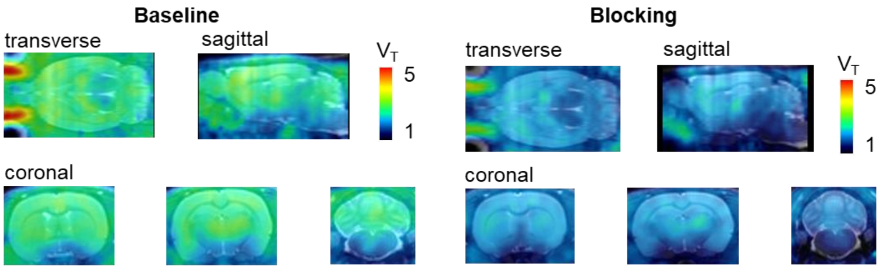

(B) Plasma input curve and time-activity curves of [^{18}F]**4** in brain regions

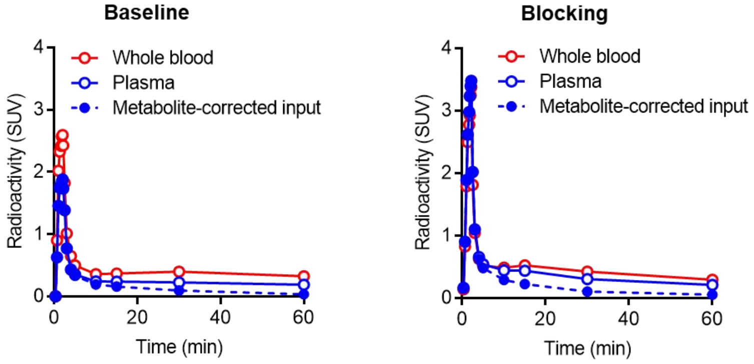

(C) Distribution volume (V_T) comparison

Regions	V_T (mL/cm^3) Baseline	V_T (mL/cm^3) Blockade	% of decrease
Cerebral cortex	2.86 ± 0.01	2.04 ± 0.25	29
Striatum	2.87 ± 0.05	2.10 ± 0.23	27
Thalamus	2.69 ± 0.20	1.97 ± 0.23	27
Hippocampus	2.74 ± 0.09	2.00 ± 0.23	27
Cerebellum	2.55 ± 0.15	1.75 ± 0.24	31

Figure S2. PET study of [^{18}F]**4** with blood sampling. (A) Representative PET images; (B) Plasma input curve and time-activity curves of [^{18}F]**4** in brain regions; (C) Distribution volume (V_T) comparision.

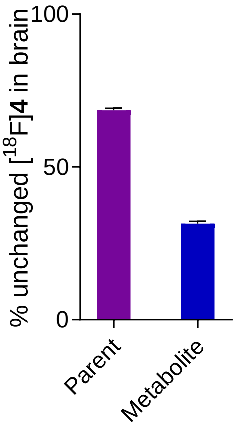

Figure S3. Metabolic analysis of [^{18}F]**4** in mouse brain at 30 min post tracer injection.

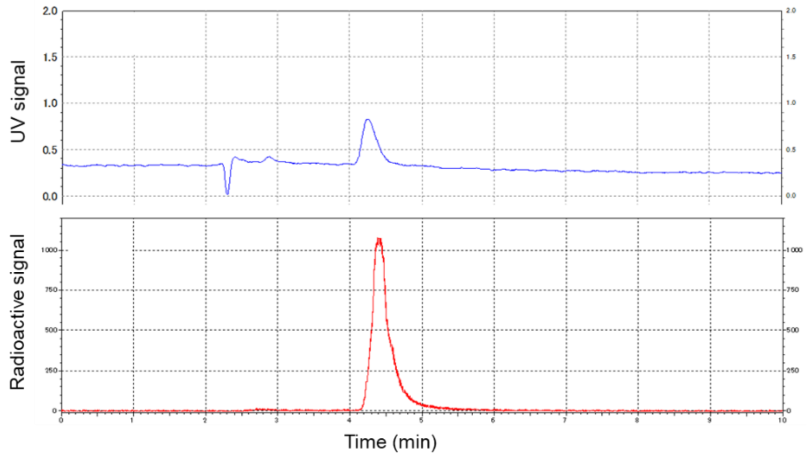

Figure S4. HPLC traces for [^{11}C]**3**.

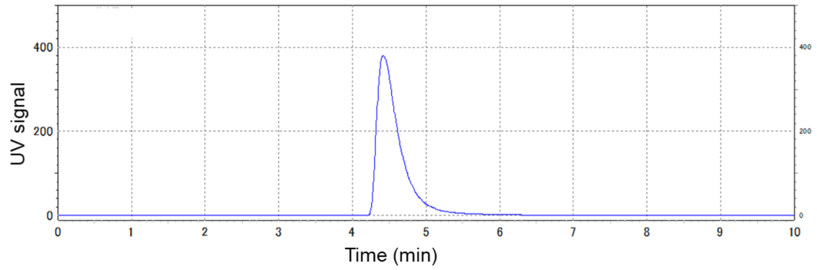

Figure S5. HPLC trace for **3**.

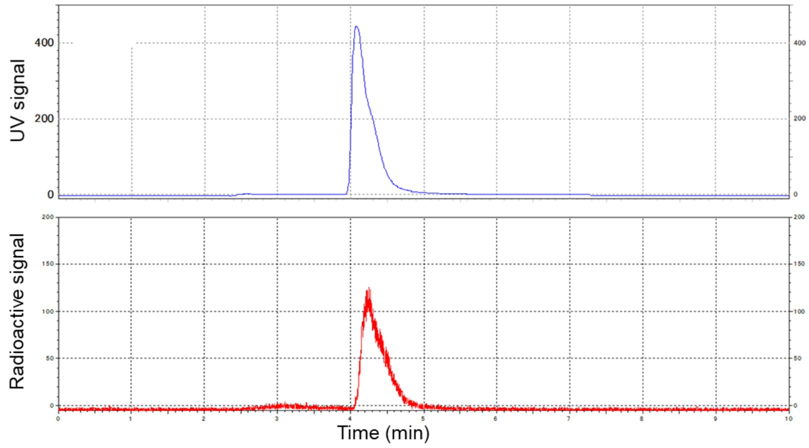

Figure S6. HPLC traces for [^{11}C]**3** co-injected with **3**.

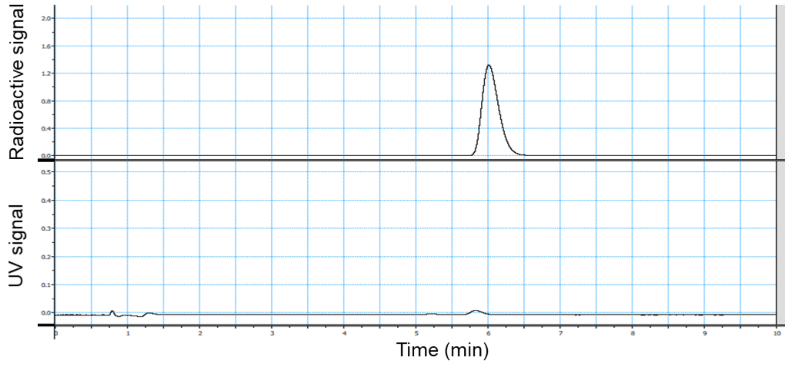

Figure S7. HPLC traces for [^{18}F]**4**.

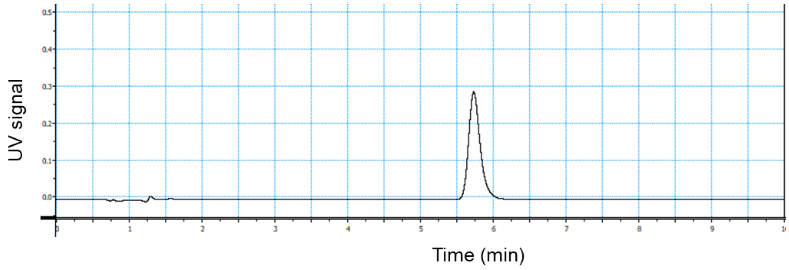

Figure S8. HPLC trace for **4**.

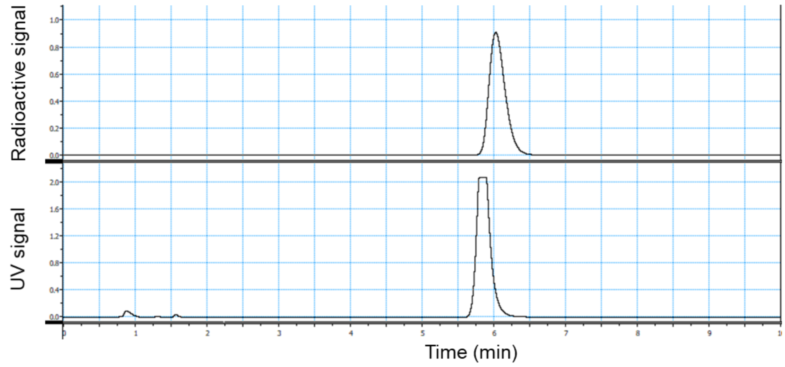

Figure S9. HPLC traces for [^{18}F]**4** co-injected with **4**.

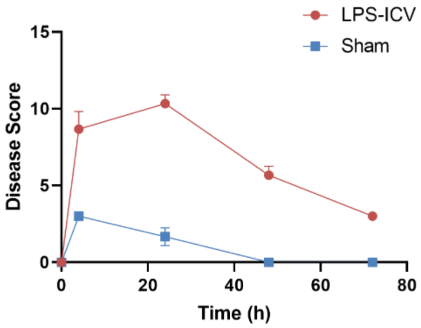

Figure S10. Disease scores of the LPS-induced mouse model every 72 hours (n = 4).

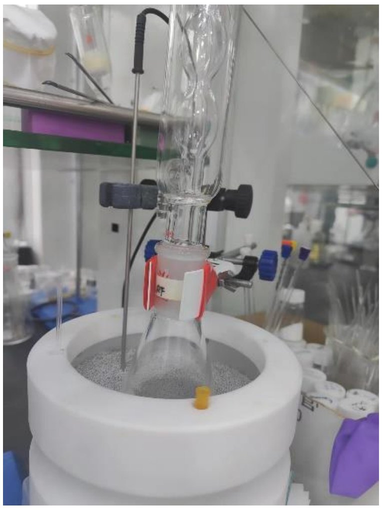

Figure S11. All heating reactions were heated by a metal sand bath (WATTCAS, LAB-500).

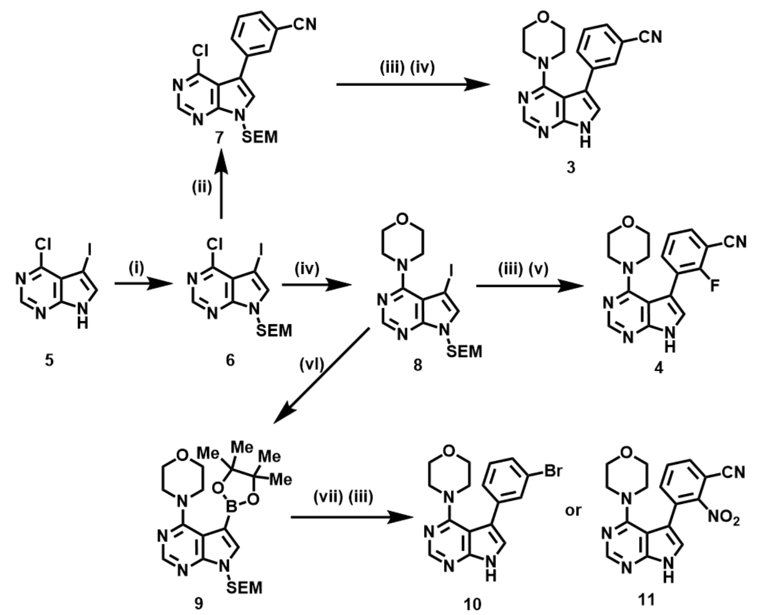

Conditions: (i) SEM-Cl, NaH, THF, rt, 3 h; 57% yield; (ii) (3-cyanophenyl)boronic acid, Pd(dppf)Cl_2, K_2CO_3, DME, H_2O, 90 ºC, 3 h; 65% yield; (iii) TFA, rt, 2 h; (iv) morpholine, DIPEA, n-BuOH, reflux, 18 h; (iii) aryl-boronic acid, Pd(dppf)Cl_2, K_2CO_3, EtOH, H_2O, 100 °C, 18 h; (v) (3-cyano-2-fluorophenyl)boronic acid, $Pd(PPh_3)_2Cl_2$ K_2CO_3, EtOH, H_2O, 100 ºC, 18 h; (vi) bis(pinacolato)diboron, $Pd_2(dba)_3$, XPhos, TEA, dioxane, 95 °C, 18 h; 83% yield; (vii) $Pd(PPh_3)_4$, Na_2CO_3, dioxane/H_2O, 120 °C, 1-bromo-3-iodobenzene for **10**, 3-chloro-2-nitrobenzonitrile for **11**.

Scheme S1. Synthesis of standard LRRK2 inhibitors **3&4** and their radiolabeling precursors **10&11**.[1, 2]

Analytical HPLC method: Radiochemical and chemical purity of [^{11}C]**3** was measured by analytical HPLC (COSMOSIL Cholester column (4.6 x 250 mm, 5 µm) using a mobile phase of CH_3CN / 0.1 M NH_4OAc (60/40) at a flow rate of 1.0 mL/min. The identity of [^{11}C]**3** was confirmed by the co-injection with unlabeled **3**. Radiochemical and chemical purity of [^{18}F]**4** was measured by analytical HPLC (Gemini NX-C18 column (3 x 150 mm, 5 µm) using a mobile phase of CH_3CN / 0.1 M AMF (30/70) at a flow rate of 0.8 mL/min. The identity of [^{18}F]**4** was confirmed by the co-injection with unlabeled **4**.

Animal oversite group statement: The PET imaging studies in SD rats were performed at the National Institutes for Quantum and Radiological Science and Technology; The PET imaging studies in mouse models and biodistribution studies in CD-1 mice were performed at Massachusetts General Hospital and Emory University.

The standard LRRK2 inhibitors **3&4** and their radiolabeling precursors **10&11** were synthesized according to the procedures as described in previous literatures.[1, 2]

Synthesis of 3-(4-morpholino-7*H*-pyrrolo[2,3-d]pyrimidin-5-yl)benzonitrile (3). Step 1. A solution of 3-(4-chloro-7-((2-(trimethylsilyl)ethoxy)methyl)-7*H*-pyrrolo[2,3-d]-pyrimidin-5-yl)benzonitrile (**7**) (1.0 mmol) in trifluoroacetic acid (2.5 mL) was

stirred at room temperature for 2 h. The reaction mixture was concentrated in vacuo and then taken up in methanol (10 mL) and adjusted to pH >12 by addition of solid potassium carbonate. Solvent was removed in vacuo, and the residue was mixed with water (10 mL). The resulting solid was isolated via filtration and washed with water, providing 3-(4-chloro-7H-pyrrolo[2,3-d]pyrimidin-5-yl)benzonitrile as a white solid.

Step 2. Morpholine (1.0 mmol) and N,N-diisopropylethylamine (1.0 mmol) were added to a solution of the above-obtained product in n-butanol (10 mL), and the reaction mixture was heated at reflux for 3 h. Solvents were removed in vacuo, and the residue was purified using chromatography on silica gel (eluent, 1:1 ethyl acetate/petroleum ether). Subsequent recrystallization from ethyl acetate and tert-butyl methyl ether afforded the product as a white solid. ^{1}H NMR (600 MHz, DMSO) δ 12.33 (s, 1H), 8.40 (s, 1H), 8.00 (s, 1H), 7.91 – 7.86 (m, 1H), 7.78 – 7.73 (m, 1H), 7.72 – 7.64 (dd, *J* = 16.4, 8.7 Hz, 2H), 3.49 – 3.42 (m, 4H), 3.17 – 3.11 (m, 4H).

Synthesis of 2-(fluoro)-3-(4-morpholino-7H-pyrrolo[2,3-d]pyrimidin-5-yl)benzonitrile (4). Step 1. To a solution of **8** (1.1 mmol) and (3-cyano-2-fluorophenyl)boronic acid (1.31 mmol) in a mixture of ethanol and water (4:1, 10 mL) were added dichlorobis(triphenylphosphine)palladium(II) (58 μmol) and potassium carbonate (3.23 mmol). After degassing and purging with nitrogen three times, the reaction mixture was allowed to stir at 100 °C for 18 h. Then the reaction mixture was concentrated in vacuo, and the residue was purified by silica gel chromatography with ethyl acetate/petroleum ether (1:1) as eluent to deliver the desired product 2-fluoro-3-(4-morpholino-7-((2-(trimethylsilyl)ethoxy)methyl)-7*H*-pyrrolo[2,3-d]pyrimidin-5-yl)benzonitrile as a yellow solid.

Step 2. A solution of 2-fluoro-3-(4-morpholino-7-((2-(trimethylsilyl)ethoxy)methyl)-7*H*-pyrrolo[2,3-d]pyrimidin-5-yl)benzonitrile (0.48 mmol) was treated with trifluoroacetic acid (TFA, 5 mL) at room temperature for 2 h. The reaction mixture was then concentrated in vacuo, and the resulting yellow oil was re-dissolved in 5 mL of MeOH. The pH of the reaction mixture was regulated above 12 using solid K_2CO_3. After stirring at room temperature for 30 min, the reaction mixture was filtered and concentrated under reduced pressure. The residue was purified by preparative reverse HPLC (column, Agella Venusil ASB C18, 5 μm; mobile phase A, 0.225% formic acid in water; mobile phase B, acetonitrile; eluent, 13% B) to deliver the desired product as

a white solid. ^{1}H NMR (300 MHz, DMSO) δ 12.39 (s, 1H), 8.39 (s, 1H), 7.93 – 7.78 (m, 2H), 7.63 (s, 1H), 7.57 – 7.48 (m, 1H), 3.40 – 3.34 (m, 4H), 3.15 – 3.10 (m, 4H).

Synthesis of 3-(4-Morpholino-7H-pyrrolo[2,3-d]pyrimidin-5-yl)-2-nitrobenzonitrile (11). The synthetic procedure was described previously with minor revision herein.[2] Step 1. To a solution of aryl halide (1.0 mmol) and **9** (1.2 mmol) in 1,4-dioxane (5 mL) and water (1 mL) was added tetrakis(triphenylphosphine)palladium(0) (0.1 mmol) and sodium carbonate (3.0 mmol). The reaction mixture was heated at 120 °C under microwave irradiation for 15 min, then diluted with water and extracted with ethyl acetate (3 × 50 mL). The combined organic layers were dried over sodium sulfate, filtered, and concentrated in vacuo. The residue was purified via chromatography on silica gel (eluent, 1:1 ethyl acetate/petroleum ether) to provide the product as a yellow solid.

Step 2. The product (0.5 mmol) obtained in Step 1 was dissolved in 5 mL of TFA at room temperature. After stirring at the same temperature for 2 h, the reaction mixture was concentrated to remove the volatile and the residue was re-dissolved in 5 mL of MeCN. The pH of the mixture was regulated above 12 using solid K_2CO_3. After stirring at room temperature for 30 min, the reaction mixture was filtered and concentrated under reduced pressure. The residue was purified by preparative reverse HPLC (column, Phenomenex Gemini C18, 8 μm; mobile phase A, ammonia in water, pH 10; mobile phase B, acetonitrile; gradient, 10−50% B) afforded the product as a yellow solid. ^{1}H NMR (300 MHz, DMSO) δ 8.37 (s, 1H), 8.19 – 8.12 (m, 1H), 7.97 – 7.91 (m, 2H), 7.49 (s, 1H), 3.37 – 3.31 (m, 1H), 3.11 – 3.06 (m, 1H).

2. NMR Spectra

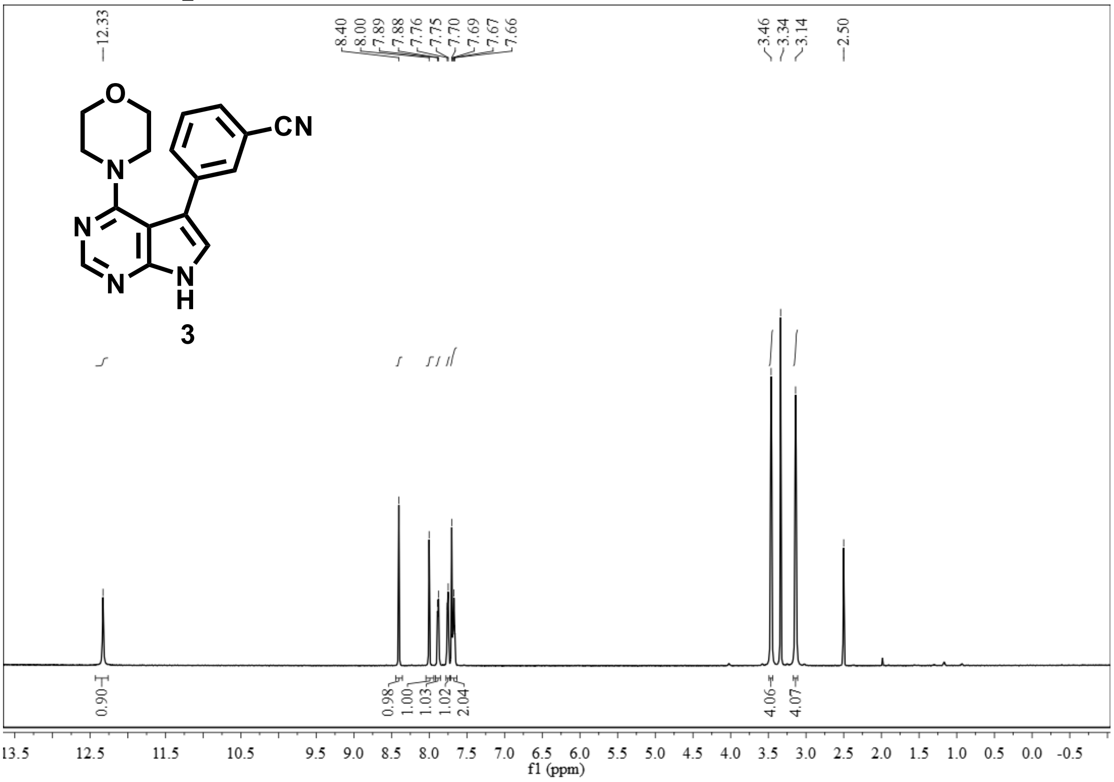

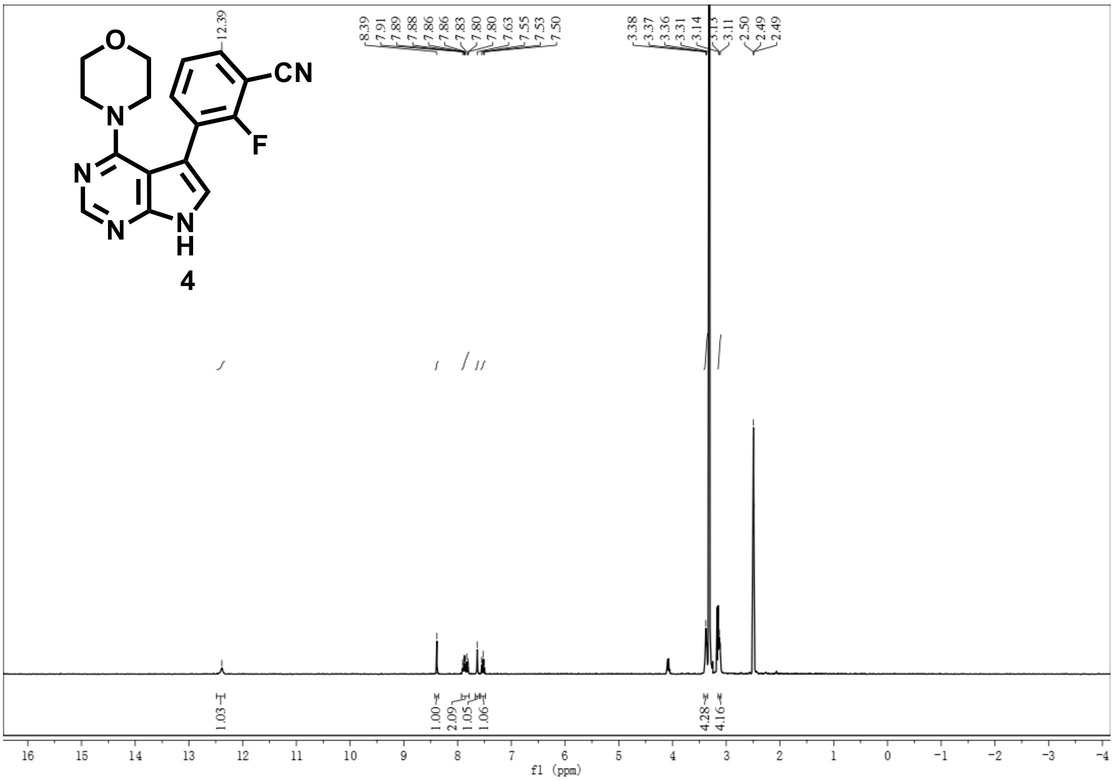

3. References

(1) Chen, Z.; Chen, J.; Chen, L.; Yoo, C.-H.; Rong, J.; Fu, H.; Shao, T.; Coffman, K.; Steyn, S. J.; Davenport, A. T.; Daunais, J. B.; Haider, A.; Collier, L.; Josephson, L.; Wey, H.-Y.; Zhang, L.; Liang, S. H. Imaging leucine-rich repeat kinase 2 in vivo with ^{18}F-labeled positron emission tomography ligand. *J. Med. Chem.* **2023,** 66, 1712-1724.

(2) Henderson, J. L.; Kormos, B. L.; Hayward, M. M.; Coffman, K. J.; Jasti, J.; Kurumbail, R. G.; Wager, T. T.; Verhoest, P. R.; Noell, G. S.; Chen, Y.; Needle, E.; Berger, Z.; Steyn, S. J.; Houle, C.; Hirst, W. D.; Galatsis, P. Discovery and preclinical profiling of 3-[4-(morpholin-4-yl)-7*H*-pyrrolo[2,3-d]pyrimidin-5-yl]benzonitrile (PF-06447475), a highly potent, selective, brain penetrant, and in vivo active LRRK2 kinase inhibitor. *J. Med. Chem.* **2015,** 58, 419-432.
